# A Unique Case of Localized Palmar Erythema Annulare Centrifugum

**DOI:** 10.7759/cureus.25718

**Published:** 2022-06-07

**Authors:** Griffin Clyatt, John Cole

**Affiliations:** 1 Osteopathic Medicine, Philadelphia College of Osteopathic Medicine, Moultrie, USA; 2 Dermatology, Cole Dermatology LLC, Valdosta, USA

**Keywords:** annular patches and plaques, gyrate erythemas, hydrochlorothiazide, erythema annulare centrifugum, figurate erythemas

## Abstract

Figurate erythemas (FE) are a group of diseases defined by the presence of annular or arciform erythematous skin lesions that can occur anywhere on the body. Four different types of figurate erythemas have been described: erythema annulare centrifugum (EAC), erythema gyratum repens (EGR), erythema migrans, and erythema marginatum. While each of these presents with similar morphologies, the clinical and histopathological features are often unique and can aid in the diagnosis. Although the majority of the figurate erythemas are idiopathic, some may be associated with infections, stress, or other diseases. We present a novel case of recurrent figurate erythema in a 27-year-old male that appeared to be exacerbated by hydrochlorothiazide (HCTZ) and social stressors.

## Introduction

Figurate erythemas (FE) can be divided into four classic categories: erythema annulare centrifugum (EAC), erythema gyratum repens, erythema migrans, and erythema marginatum. The diagnosis of FE can be challenging due to its variation in presentation, with similar morphologies and a broad disease differential. Skin biopsy and histopathological correlation are the gold standards for the diagnosis of these lesions, although clinical and histological features provide a clue to the correct diagnosis.

## Case presentation

A 27-year-old male medical student presented to the dermatology clinic with the chief complaint of a bilateral, non-pruritic, palmar rash of approximately one-year duration. The annular plaques had inflamed edges with central clearing and focal areas of desquamation; no similar lesions were noted elsewhere. Past medical history included attention deficit hyperactivity disorder (ADHD) diagnosed at age 15 and stage 1 essential hypertension diagnosed at age 26. He also stated that he had significant stressors in his life recently that involved societal and familial matters. Dermatological family history was significant for scalp psoriasis on his paternal side. Medications included hydrochlorothiazide (HCTZ) 12.5 mg/day for the past year and Adderall 20 mg/day. 

Approximately four months prior to his dermatology visit, the patient was evaluated by his primary care provider and was empirically diagnosed with bilateral tinea manuum and treated with a course of oral terbinafine without improvement. During his initial dermatology visit, a biopsy was discussed but deferred. For the presumed inflammatory eruption on the palms, empiric treatment was initiated with betamethasone dipropionate 0.05% cream. One week later, the patient reported that the lesions were responding to the topical steroid but still opted to proceed with biopsy for a definitive diagnosis.

The pathology report showed "subtle superficial perivascular dermatitis with minimal epidermal spongiosis." The microscopic examination showed "an infiltrate of inflammatory cells consisting mostly of lymphocytes present around vessels of the superficial plexus." It was noted that Treponema pallidum immunostaining and periodic acid-Schiff (PAS) stain were both negative.

**Figure 1:**
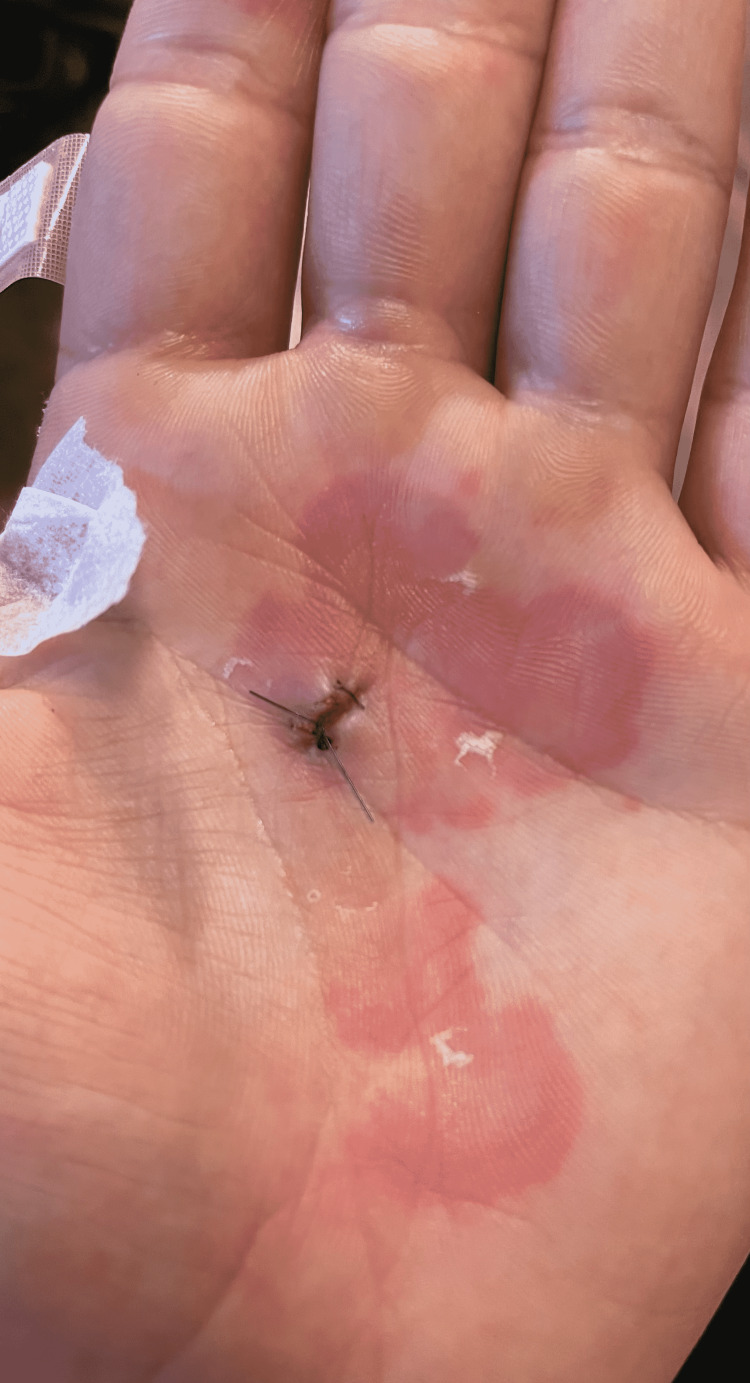
Annular lesions post-biopsy Source: Original upload Credit: Griffin Clyatt, John Cole MD

**Figure 2:**
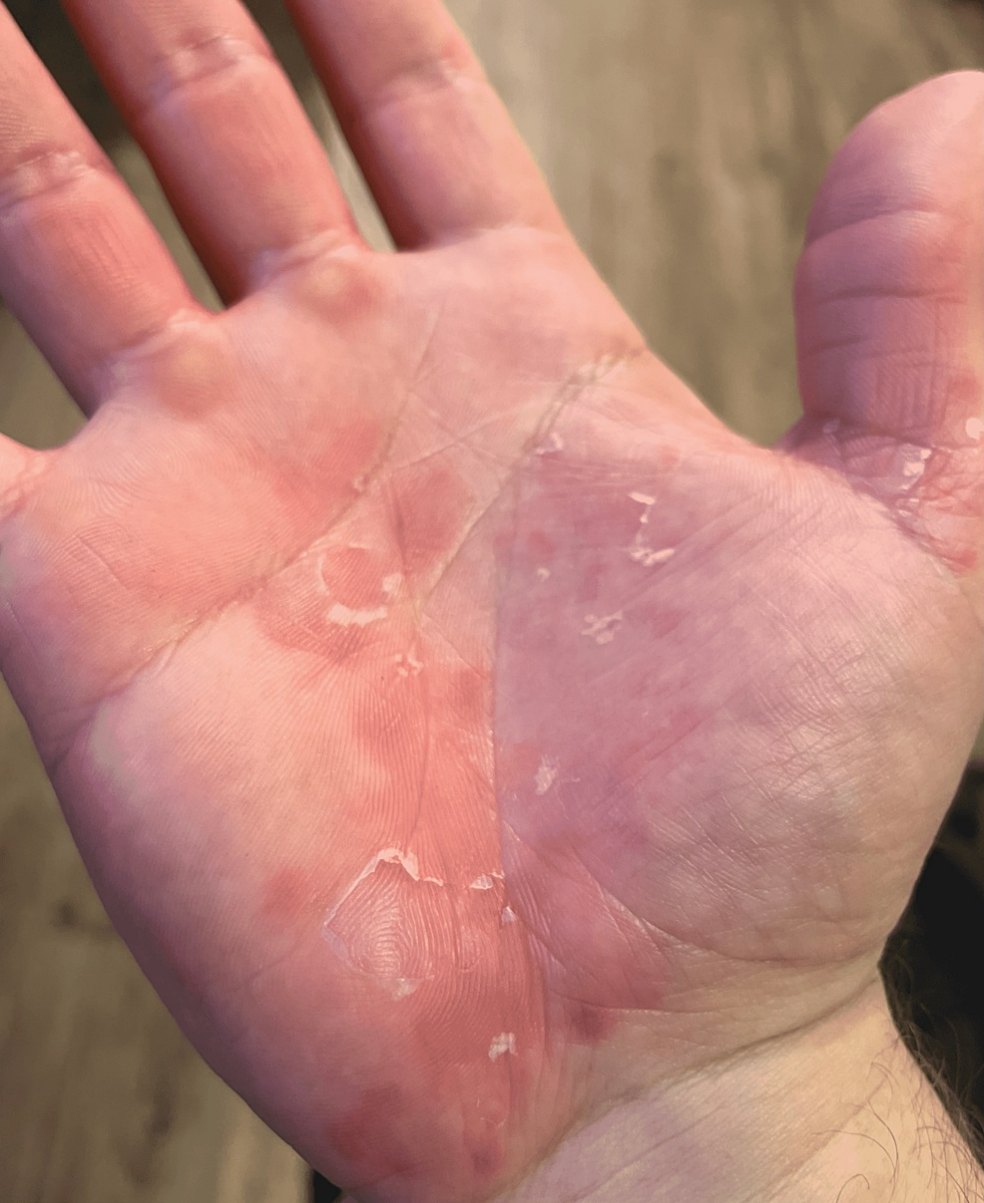
Annular lesions with superficial scale Source: Original upload Credit: Griffin Clyatt, John Cole MD

**Figure 3:**
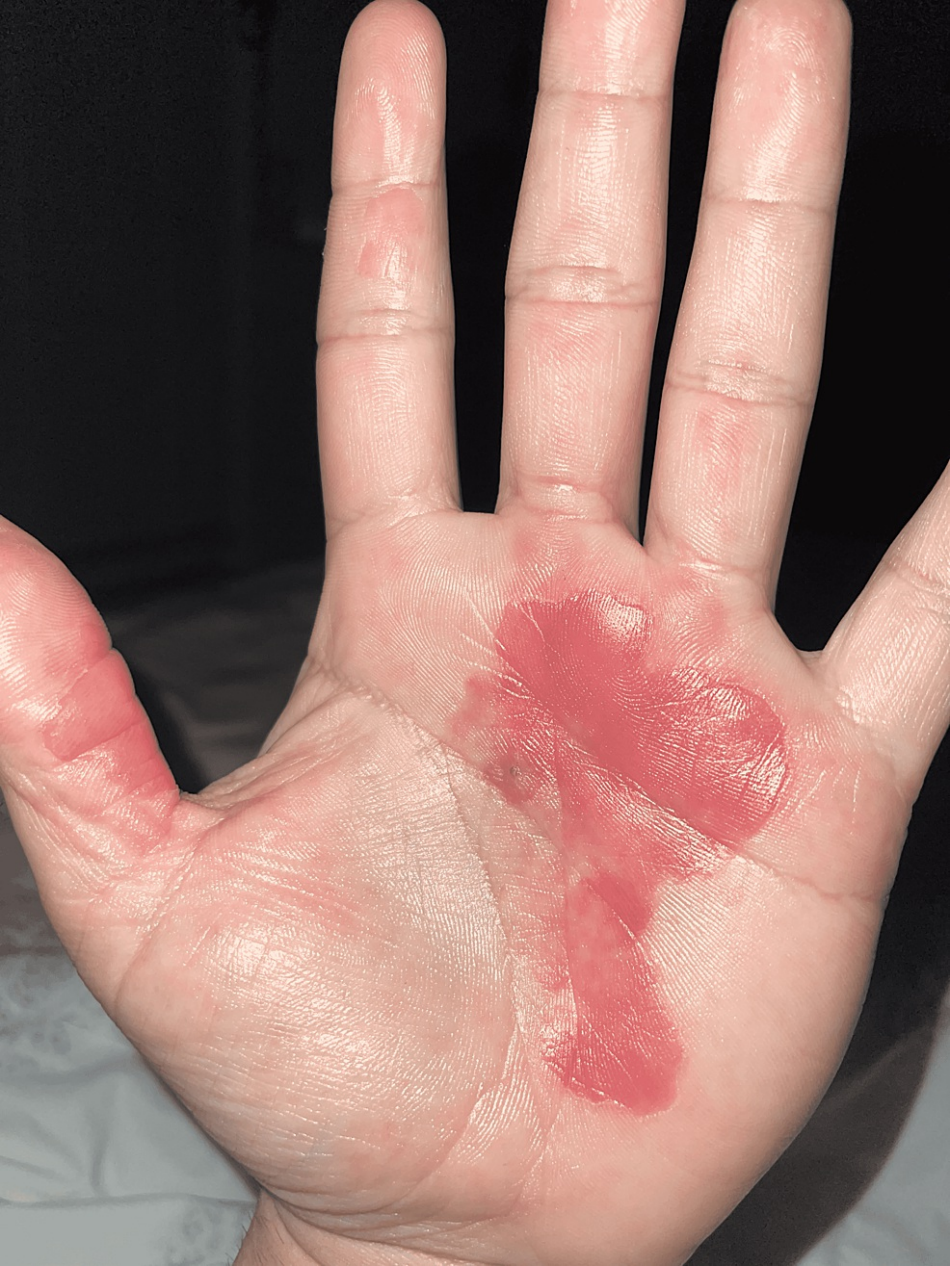
Inflammation of the previous lesions shortly after the patient took hydrochlorothiazide Source: Original upload Credit: Griffin Clyatt, John Cole MD

At suture removal, the patient was prescribed a 12-day oral prednisone taper (40 mg for three days, 30 mg for three days, 20 mg for three days, and 10 mg for three days) because the rash was persistent despite topical steroid use. The images in Figures 1-2 demonstrate the erythematous annular lesions and erythematous lesions with scaling, respectively. Approximately five days before the completion of oral prednisone, the patient began noting some potential association with flaring of his palms shortly after taking his daily HCTZ (Figure 3). Subsequently, he discontinued the diuretic and noted no new development of lesions. His lesions resolved and remained clear for approximately eight weeks, but then he noted the recrudescence of a few isolated inflamed macules on the right palm. He also reported some episodic outbreaks which responded well to the topical betamethasone and seemed to flare around times of increased stress.

## Discussion

Based on the clinical presentation, the differential diagnosis included a type of figurate erythema (FE), erythema multiforme, secondary syphilis, dermal hypersensitivity, spongiotic dermatitis, and tinea manuum. The majority of these diagnoses were ruled out due to the patient's past medical history, clinical findings, and biopsy results from the left ulnar palm. We will briefly review the classic FEs and note some of the associations reported to occur with EAC, the most common FE.

Figurate erythemas, also known as gyrate erythemas, typically begin as pink macules or papules that expand centrifugally with a central clearing, creating the classic annular or arciform appearance. With the exception of erythema marginatum, the lesions can be non-scaling or scaling and tend to persist for more than 24 hours. The erythema and cutaneous inflammation are thought to be due to the production of inflammatory cytokines, including TNF-𝞪 and IL-1 [1].

**Figure 4:**
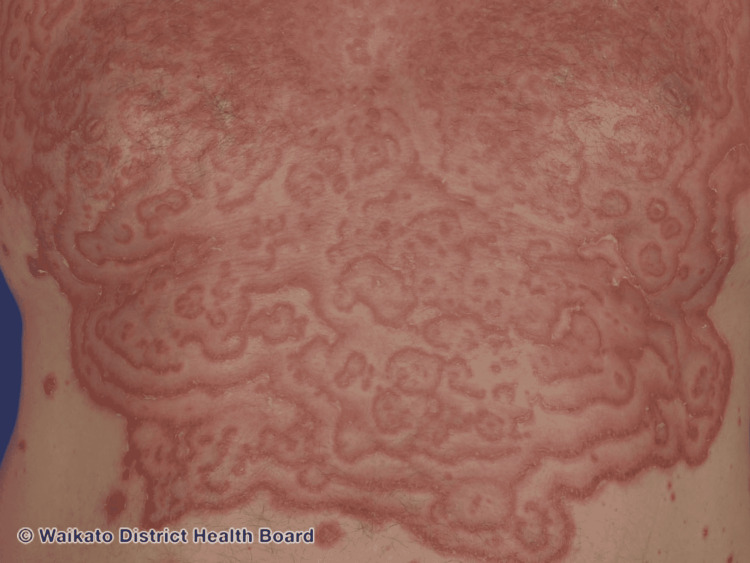
Erythema gyratum repens-like appearance in pemphigus foliaceus: Wood-grained appearance and Zebra-like pattern Credit: Dermnet Source: https://dermnetnz.org/topics/erythema-gyratum-repens License: https://creativecommons.org/licenses/by-nc-nd/3.0/nz/legalcode

Erythema gyratum repens (EGR) is characterized as a migratory erythematous lesion composed of concentric rings that have a wood-grain appearance. Figure 4 shows the classic morphology of this rare FE. EGR is typically a paraneoplastic process and is most often associated with cancers of the lungs, breasts, stomach, or esophagus. EGR may represent a cross-reaction between cutaneous antigens and tumor antigens. In lesions of EGR, immunohistochemical staining has demonstrated the presence of IgG and C3 deposits on the basement membrane of the skin. The lesions develop scales at their leading edges but spread much faster than EAC and often develop on top of each other, leading to the wood-grain appearance or a zebra-like pattern. Histology is nonspecific and can include focal parakeratosis, moderate patchy spongiosis, and perivascular lymphohistiocytic infiltrate. Sometimes melanophages and eosinophils can be seen in the dermis [1]. EGR was ruled out given the obvious absence of morphologic similarity as well as a review of systems negative for any evidence of systemic disease.

Erythema migrans is a distinct classical reaction to an infection with Borrelia burgdorferi, transmitted by ticks. It has a "bull’s eye" appearance and is seen as an early manifestation of Lyme disease. It is described as an annular erythema at the site of the tick bite and is seen in 60-90% of patients diagnosed with Lyme disease. Approximately 7-15 days after tick detachment, the rash can be seen as an expanding erythematous annular plaque with centrifugal clearing and central erythema that classically resembles a bull’s eye. Clinically, patients typically have systemic manifestations resembling flu-like symptoms that correlate with the appearance of the rash. These symptoms may include headaches, fatigue, arthralgias, myalgias, and lymphadenopathy. The histological sample from a biopsy would correlate more with deep gyrate erythema, with eosinophils often being noted. In the dermis, the inflammatory infiltrate would typically show macrophages, CD4+ helper T cells, and CD45RO+ memory T cells. In the epidermis, multiple apoptotic keratinocytes may be seen. In addition, the Warthin-Starry stain may be able to detect spirochetes in the skin [1]. Due to the absence of a history of a tick bite and a lack of systemic symptoms, erythema migrans was ruled out. 

Erythema marginatum, also known as erythema annulare rheumaticum, is an inflammatory cutaneous reaction caused by group A β-hemolytic streptococci and is seen in rheumatic fever (RF). RF involves a triad of arthritis, fever, and carditis. The etiology of this dermatological reaction is thought to be due to antigenic mimicry in which the bacterial surface antigens lead to cutaneous sensitization [1]. This cutaneous sensitization then leads to an immunologic cross-reaction and manifestation of the lesions. The skin manifestations can include subcutaneous nodules and erythematous macules that gradually spread peripherally. These nodules and macules can lead to plaques or patches, but there is no associated scaling. The common locations include the proximal extremities, trunk, and axillae. These lesions are seen primarily in the active phase of RF. The histopathology would show an interstitial and perivascular infiltrate composed of a predominance of neutrophils [1]. In our patient’s case, due to the absence of systemic symptoms or any other features of RF, the diagnosis of erythema marginatum was excluded.

**Figure 5:**
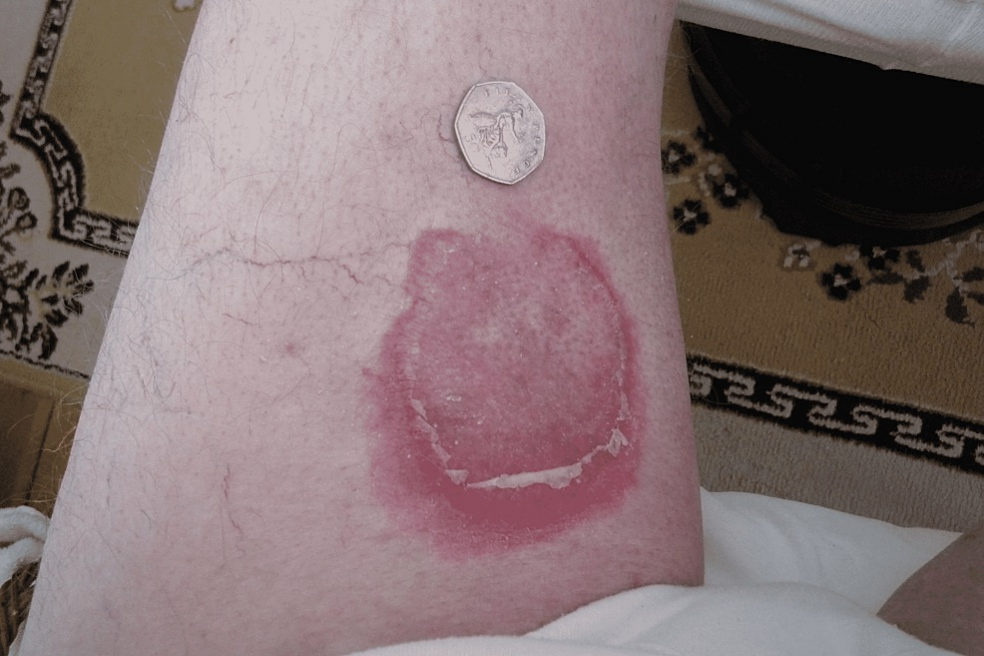
Erythema annulare centrifugum Credit: Jim France Source: https://commons.wikimedia.org/wiki/File:Erythema_annulare_centrifugum.JPG#filelinks License: https://creativecommons.org/licenses/by-sa/3.0/legalcode

Although erythema annulare centrifugum (EAC) is the most common figurate erythema, it is nonetheless rare. EAC is a rare cutaneous eruption that is characterized by erythematous, annular patches, and plaques that begin centrally as papules and expand peripherally with central clearing. Figure 5 shows the annular ring-like plaque of EAC and the superficial fine scale that often accompanies the lesion. There is no clear etiology of EAC, and most cases are considered idiopathic, but it has been associated with certain drugs, including diuretics (hydrochlorothiazide), non-steroidal anti-inflammatory drugs, antimalarials, finasteride, gold, and amitriptyline. Other underlying conditions, including autoimmune diseases and malignancies, have been reported in association with EAC [1]. Most often, in superficial lesions of EAC, the findings are nonspecific on pathology and show mild spongiosis, microvesiculation with associated focal parakeratosis, and mild superficial perivascular lymphohistiocytic infiltrate. The perivascular lymphocytes on histology show a "coat sleeved" appearance, tightly aggregating around the vasculature. In the deeper lesions of EAC, the epidermis may appear normal, and the perivascular lymphocytes appear mostly in the middle and lower dermis [1]. In this patient's case, the microscopic description of the biopsy showed an "infiltration of inflammatory cells consisting mostly of lymphocytes present around vessels of the superficial plexus with minimal epidermal spongiosis." This microscopic description can be seen in the superficial lesions of EAC. Our patient's biopsy demonstrated perivascular lymphocytes around the superficial plexus. Although this finding is nonspecific, it is often noted in the superficial type of EAC. Both the clinical and microscopic findings are compatible with EAC, despite the lack of definitive pathologic findings, which are seen more often in deeper-seated lesions.

Numerous triggers have been linked to the development of EAC, including food, medications, infections, and underlying neoplastic or autoimmune processes. Specific drugs associated with EAC include finasteride, hydroxychloroquine, NSAIDs, and amitriptyline. Goette and Beatrice [2] reported a case in which HCTZ was associated with the development of EAC after the drug caused interstitial nephritis. Although the correlation between HCTZ use and the occurrence of EAC is hypothesized to be mediated through nephrogenic involvement, the precise mechanism of action remains unclear.

Stress has also been reported to be associated with EAC. Ibrahim et al. [3] published a case report of a 36-year-old male under significant psychological stress who developed a biopsy-proven EAC. The patient’s skin lesion regressed without medical treatment after the stressor was eliminated. Our patient also informed us of his stress during medical school, which correlated to the flaring of his palmar rash.

Treatment for FEs involves establishing the diagnosis and treating the underlying cause of the disease, including eliminating possible offending medications or other triggers. When there is no clear etiology, treatment with superpotent corticosteroids can decrease the inflammatory process, thus slowing lesion progression. If the lesions are pruritic, topical antipruritics and/or antihistamines can be prescribed for symptomatic relief. For severe diseases, systemic corticosteroids are the mainstay of treatment [1].

## Conclusions

We believe highlighting this case of EAC can help primary care and specialist physicians alike expand their knowledge of an uncommon skin condition. Expanding the differential diagnosis to include EAC before initiating treatment can have positive impacts on patient safety and healthcare costs. In this case, for example, initial treatment with an oral antifungal for an inflammatory condition could have led to an undesired medication side effect. Additionally, inappropriate use of medications contributes to increased healthcare expenditures by patients and their insurers. 

Although our patient’s biopsy findings were not pathognomonic for EAC, they were consistent with this diagnosis. Nonspecific biopsy results are not uncommon in the setting of superficial EAC, which often lacks the more obvious histologic findings of deeper-seated lesions. Furthermore, the patient had already started treatment with a topical steroid prior to deciding to proceed with biopsy, which could have minimized some of the inflammatory processes that may otherwise have been present. Regardless, correlation of the clinicopathologic data makes EAC the most likely diagnosis. In cases of EAC in which the rash is determined to be idiopathic, symptomatic control is the mainstay of treatment. Although recurrent or longer-lasting cases of EAC have been documented, fortunately, most cases are self-limited, resolving in about a year.
